# Whole genome sequencing-based classification of human-related *Haemophilus* species and detection of antimicrobial resistance genes

**DOI:** 10.1186/s13073-022-01017-x

**Published:** 2022-02-09

**Authors:** Margo Diricks, Thomas A. Kohl, Nadja Käding, Vladislav Leshchinskiy, Susanne Hauswaldt, Omar Jiménez Vázquez, Christian Utpatel, Stefan Niemann, Jan Rupp, Matthias Merker

**Affiliations:** 1 Molecular and Experimental Mycobacteriology, Research Center Borstel, Borstel, Germany; 2 German Center for Infection Research (DZIF), Partner Site Hamburg-Lübeck-Borstel-Riems, Hamburg, Germany; 3 Department of Infectious Diseases and Microbiology, University Hospital Schleswig-Holstein, Lübeck, Germany; 4 German Center for Infection Research (DZIF), TTU HAARBI, Lübeck, Germany; 5 Evolution of the Resistome, Research Center Borstel, Borstel, Germany

**Keywords:** Whole genome sequencing, *Haemophilus*, *H. influenzae*, *H. haemolyticus*, Pangenome-wide association study, Identification, Molecular differentiation, Antibiotic resistance, Precision medicine

## Abstract

**Background:**

Bacteria belonging to the genus *Haemophilus* cause a wide range of diseases in humans. Recently, *H. influenzae* was classified by the WHO as priority pathogen due to the wide spread of ampicillin resistant strains. However, other *Haemophilus* spp. are often misclassified as *H. influenzae.* Therefore, we established an accurate and rapid whole genome sequencing (WGS) based classification and serotyping algorithm and combined it with the detection of resistance genes.

**Methods:**

A gene presence/absence-based classification algorithm was developed, which employs the open-source gene-detection tool SRST2 and a new classification database comprising 36 genes, including capsule loci for serotyping. These genes were identified using a comparative genome analysis of 215 strains belonging to ten human-related *Haemophilus* (sub)species (training dataset). The algorithm was evaluated on 1329 public short read datasets (evaluation dataset) and used to reclassify 262 clinical *Haemophilus* spp. isolates from 250 patients (German cohort). In addition, the presence of antibiotic resistance genes within the German dataset was evaluated with SRST2 and correlated with results of traditional phenotyping assays.

**Results:**

The newly developed algorithm can differentiate between clinically relevant *Haemophilus* species including, but not limited to, *H. influenzae*, *H. haemolyticus*, and *H. parainfluenzae*. It can also identify putative haemin-independent *H. haemolyticus* strains and determine the serotype of typeable *Haemophilus* strains. The algorithm performed excellently in the evaluation dataset (99.6% concordance with reported species classification and 99.5% with reported serotype) and revealed several misclassifications. Additionally, 83 out of 262 (31.7%) suspected *H. influenzae* strains from the German cohort were in fact *H. haemolyticus* strains, some of which associated with mouth abscesses and lower respiratory tract infections*.*

Resistance genes were detected in 16 out of 262 datasets from the German cohort. Prediction of ampicillin resistance, associated with *bla*_TEM-1D_, and tetracycline resistance, associated with *tetB*, correlated well with available phenotypic data.

**Conclusions:**

Our new classification database and algorithm have the potential to improve diagnosis and surveillance of *Haemophilus* spp. and can easily be coupled with other public genotyping and antimicrobial resistance databases. Our data also point towards a possible pathogenic role of *H. haemolyticus* strains, which needs to be further investigated.

**Supplementary Information:**

The online version contains supplementary material available at 10.1186/s13073-022-01017-x.

## Background

Members of the genus *Haemophilus* are small, pleomorphic gammaproteobacteria that are characterized by their dependency on the blood factors haemin (X-factor) and/or nicotinamide adenine dinucleotide (V-factor) for growth [1, 2]. The genus *Haemophilus* includes 10 (sub)species that exclusively or predominantly colonize and infect humans [3]. Bacteria belonging to the species *H. influenzae* are commonly found as commensal in the human nasopharynx but can also be pathogenic, especially for susceptible populations such as premature infants, elderly adults, and people with compromised immune systems [4, 5]. In particular, *H. influenzae* serotype b (Hib) strains were one of the major causes of bacterial meningitis in children worldwide until the introduction of an effective conjugate vaccine [6, 7]. Since then, non-typeable (i.e., non-encapsulated) *H. influenzae* (NTHi) strains have become the most common cause of *H. influenzae* related invasive disease, exacerbations of chronic obstructive pulmonary disease and localized infections such as otitis media, pneumonia, sinusitis, bronchitis, and conjunctivitis [4, 8, 9]. Furthermore, the global expansion of ampicillin resistant *H. influenzae* strains led to the WHO classification of a priority pathogen for research and development of new antibiotics [10]. Other species, such as *H. haemolyticus* and *H. parainfluenzae* have been considered as harmless respiratory tract commensals; however, evidence is accumulating that also strains of these species should be considered as opportunistic pathogens with the potential of causing a wide range of infections [8, 11, 12]. Also *H. para(phro)haemolyticus*, *H. pittmaniae*, and *H. sputorum* have been linked to different diseases but are less frequently detected [13, 14]. Other *Haemophilus* (sub)species include *H. (haemolyticus) quentini* strains, a urogenital pathogen, *H. (influenzae) aegyptius* strains causing conjunctivitis and Brazilian purpuric fever [3, 15–17], and *H. ducreyi* strains, a causative agent of genital chancre and chronic skin ulceration [8, 18]. The classification of *H. paraphrohaemolyticus*, *H. aegyptius*, and *H. quentini*, which are closely related to *H. parahaemolyticus*, *H. influenzae*, and *H. haemolyticu*s, respectively, as separate species is still under debate [3, 15, 19, 20].

The diversity in pathogenicity of *Haemophilus* spp. strains and related disease types underlines the need for exact taxonomic differentiation of clinical *Haemophilus* strains. Current gold-standard methods, i.e., bacterial culture, PCR, and mass spectrometry, are challenged by similarities of genetic and phenotypic characteristics among *Haemophilus* spp. This is especially problematic for *H. haemolyticus*, which is often misclassified as *H. influenzae* or *H. parainfluenzae* [3, 21–24]. The consequences of taxonomic misclassifications are profound and affect disease diagnosis, antibiotic therapy, and evaluation of treatment efficacies [21]. In addition, misclassification biases prevalence estimations, hinders vaccine development, and masks the potential pathogenic effect of different *Haemophilus* spp. that are currently mainly considered as benign commensals. Over the past decades, whole genome sequencing (WGS) has become widely adopted for the analysis of pathogens due to its unprecedented discriminatory power and increased cost-efficiency. However, currently available WGS-based classification tools are not tailored towards the identification of (all) human-related *Haemophilus* species, do not provide subspecies/serotyping information, have large memory requirements, and/or are time-consuming.

To address this problem, we established a curated classification database and algorithm for the rapid identification of clinical human-related *Haemophilus* spp*.* direct from short read sequencing data. We evaluated the performance of this algorithm using both in silico generated and real WGS datasets downloaded from public repositories. Lastly, we used this algorithm in combination with in silico resistance prediction to resolve misclassifications and determine the horizontally acquired resistome of clinical isolates from a German cohort.

## Methods

### Strain collection

The number of datasets used for each species in the training, evaluation, and German cohort dataset are summarized in Additional file 1: Table S1.

### Training dataset: Publicly available genome assemblies (*n*=215)

The training dataset consists of whole genome data from all human-related *Haemophilus* spp. reported to date [3, 25]. All completed genomes of *H. influenzae* (*n*=68) and all *H. haemolyticus* genomes (*n*=61, including 4 complete, 3 scaffold, and 57 contig level assemblies) that were available on September 22, 2020 were downloaded from the National Center for Biotechnology Information (NCBI)/RefSeq Assembly database [26] (Additional file 2: Table S2). In addition, all available assembled genomes of *H. parainfluenzae* (*n*=41), *Haemophilus ducreyi* (*n*=17), *Haemophilus aegyptius* (*n*=5), *Haemophilus parahaemolyticus* (*n*=8), *Haemophilus paraphrohaemolyticus* (*n*=4), *Haemophilus pittmaniae* (*n*=3), *Haemophilus quentini* (*n*=3), and *Haemophilus sputorum* (*n*=5) were also downloaded from RefSeq. Reads for these 215 assemblies were simulated using dwgsim [27] with a mean coverage depth of 100 and read length of 251 bp and the following parameters: -d 500 -s 50 -r 0 -F 0 -R 0 -X 0 -y 0.

### Evaluation dataset: Publicly available sequencing data (*n*=1329)

All paired-end Illumina datasets (with two fastQ files per strain) that were available on November 12, 2020 and annotated as *H. influenzae*, *H. haemolyticus*, *H. ducreyi*, *H. parainfluenzae*, *H. parahaemolyticus*, *H. paraphrohaemolyticus*, *H. pittmaniae*, or *H. sputorum* were downloaded from the sequence read archive (SRA) (*n*=1323) [28]. In addition, all *H. parainfluenzae* datasets from bioproject PRJEB37651 (*n*=51) and all available Ion Torrent datasets (one fastQ file per strain) from *Haemophilus* spp. that were available on July 27, 2021, were downloaded from SRA (*n*=100 *H. influenzae* datasets). All datasets with estimated mean coverage depth below 15 (using the median genome length reported at the NCBI website [29]) were removed (*n*=19). Transformants created in the lab (PRJNA308311, *n*=35) and strains with the same sample name (*n*=38) were also removed. Finally, read datasets that corresponded to an assembly already included in the training dataset (and thus had the same biosample number) (*n*=48) or for which we could not retrieve an assembly (*n*=5; Only Ion Torrent data) were also excluded. Unfortunately, all available *H. sputorum* and *H. parahaemolyticus* datasets were filtered out using these selection criteria, leaving no datasets left to analyze for these two species. The only three available datasets of *Haemophilus pittmaniae* could also not be used due to low quality (>83% unidentified reads according to the NCBI taxonomy analysis tool). In total, 1329 *Haemophilus* datasets remained and constituted the “evaluation dataset” (1060 *H. influenzae*, 124 *H. haemolyticus*, 140 *H. parainfluenzae*, *2 H. paraphrohaemolyticus*, and 3 *H. ducreyi* datasets—reported species classification at SRA) (Additional file 1: Table S1 and Additional file 2: Table S2).

### German cohort dataset: bacterial isolates from patients hospitalized in Germany (*n*=262)

Between April 2008 and April 2013, different specimen types were collected from 250 in- and outpatients of the University Hospital Schleswig-Holstein, Medical Clinic Borstel and Wismar. These included blood, eye smears, wound smears, and tracheostomal samples, as well as upper respiratory tract (pharyngeal suction/smear, nose smear, and tonsillar smear) and lower respiratory tract specimens (sputum, tracheobronchial aspirates, bronchial secretion, and bronchoalveolar lavage) (Additional file 3: Table S3). The samples were taken from routine diagnostics for which the ethical vote allowed us to collect certain clinical and patient-related information on a retrospective level. The reason for the collection of the samples was based on the diagnostic workflow of the respective physicians with regard to the individual clinical symptoms and additional laboratory parameters of the patients. The patients were not included in previous studies.

### Phenotypic and molecular analysis of the German cohort

Chocolate agar plates were inoculated and incubated at 35–37°C and 5% CO_2_ and evaluated for growth after 24h and 48h. For samples that are not considered primarily sterile (e.g., respiratory specimens), oleandomycin discs were used to suppress bacteria other than *Haemophilus* spp. strains. Single colonies (morphologically) suggestive of *Haemophilus* were picked for processing (i.e., further isolation to receive a subculture or identification) and subsequent freezing. In total, 262 *Haemophilus* isolates were obtained and identified at time of diagnosis (2008–2013) as *H.* influenzae by matrix-assisted laser desorption/ionization time-of-flight mass spectrometry (MALDI-TOF MS) (Bruker MALDI Biotyper®, Germany). We also reanalyzed several strains that were still viable by current MALDI-TOF equipment (Bruker Biotyper® sirius System with Flex Control 3.4 software). Measuring and classification were performed using MBT compass software 4.1 equipped with MBT IVD library (BuildV9.0, extension V1.0). All isolates were recultivated for 24h on chocolate agar plates before extraction of genomic DNA using Qiagen Genomic-tip 100/G. DNA was sequenced in 2013 with the Illumina Miseq benchtop sequencer using 251 bp paired-end reads and Nextera XT library preparation kit according to the manufacturer’s instructions (Illumina, USA). All datasets had a minimum mean coverage depth of at least 50-fold. Quality control of the WGS data and primary taxonomic analysis was performed using fastQC [30] and kraken2 [31] with a standard database (downloaded on June 09, 2020) and a minimum base quality requirement of 15, respectively. Haemin-dependency was checked phenotypically by growing the isolates at MacFarland 0.5 in the presence of MAST®Discs ID X&V (MAST Diagnostica, Germany) for 24h at 37°C. Antimicrobial susceptibility tests were performed using minimal inhibitory concentration (MIC) test strips (bestbion, Germany) for ampicillin (β-lactam antibiotic), ampicillin-sulbactam (4 μg/mL) (β-lactam antibiotic + β-lactamase inhibitor), ceftriaxone (β-lactamase stable cephalosporin antibiotic), and tetracycline following the application guide provided by the manufacturer. All results were interpreted according to the guidelines and clinical breakpoints of the European Committee on Antimicrobial Susceptibility Testing (EUCAST, v11.0 [32]) (only available for *H. influenzae*). BBL Cefinase™ discs (Becton, Dickinson and Company, USA) were used for the detection of β-lactamase activity.

### Bioinformatic analysis

#### Assembly and annotation

Assemblies for Illumina sequencing data were made using shovill v1.1.0 [33] with trimming and downsampling to 100x coverage where applicable and using SPAdes v.3.14.0 [34] without read error correction as the assembly algorithm. If SPAdes failed to produce an assembly, SKESA v.2.3.0 [35] was used instead (Additional file 2: Table S2 and Additional file 3: Table S3).

For Ion Torrent data, assemblies were made using shovill-se v1.1.0 [36] with trimming and downsampling to 100x coverage were applicable and using SPAdes as the assembly algorithm (Additional file 2: Table S2).

Prokka v1.14.6 [37] was used to (re)annotate all assemblies using a minimum contig length of 200 and *Haemophilus* as genus.

#### Pangenome-wide association study

The *H. influenzae* and *H. haemolyticus* genomes (*n*=129) from the training dataset were re-annotated by prokka and used as input for the pangenome calculation tool Roary v.3.13.0 [38]. Default parameters were used, except for blastP identity that was set at 60% instead of 95% to prevent over-splitting [39]. The optional -s parameter of Roary was used to prevent the split of paralogues into different orthologues clusters. This was done because SRST2 [40] is also not able to make the distinction between paralogues and orthologues.

#### Phylogenetic analysis

Core gene alignments were performed with Roary v.3.13.0 [38] (default parameters, except for 60% blastP identity) using PRANK (with MAFFT) and considering only gene sequences that were present in more than 90% of the strains. FastTree v2.1.9 [41] was used to calculate approximately-maximum-likelihood phylogenetic trees based on these core gene alignments. The trees were midpoint rooted using Figtree v1.4.4 [42] and visualized using iTOL v6.1.2 [43]. Additional annotation was added using inkscape [44].

#### Reference databases

##### Taxonomic classification database

The new taxonomic classification database that was developed in this study comprises 74 alleles for 36 marker genes, including genes for serotyping, and is freely available as a SRST2-compatible multi-fasta file on Github (project name HaemoSeq, https://github.com/ngs-fzb/HaemoSeq) [45]. Details about the marker genes/alleles are summarized in Additional file 4: Table S4. The nucleotide sequences of the marker genes for (sub)species classification were extracted from publicly available genomes using BioNumerics v7.6. For region I and III capsule genes, the alleles from *H. influenzae* serotype f strain KR494 were used [46] as well as those from a *H. influenzae* serotype c strain to take into account the sequence divergence between the two big serotype subclades described previously [47]. For region II capsule genes of typeable *H. influenzae* strains, the six concatenated sequences (*acs*, *bcs*, *ccs*, *dcs*, *ecs*, and *fcs*) included in the seq_typing repository [48] were used as references. Lastly, region II alleles for the three main serotypes of *H. sputorum* and *H. parainfluenzae* [25] were extracted from two publicly available genomes (HSPU-1 from AFNK01 and HSPU-2 from QEPN01) or a SPAdes assembly (HPAR-1) and included in the database. As HPAR-2 is very closely related to HSPU-2 [25], this was not included as separate allele.

##### Resistance database

The public resistance database ARGannot_r3 (last update October 27, 2018) was downloaded from the SRST2 repository [49]. This database contains 557 resistance genes for 12 antibiotic classes.

##### Multi-locus sequence typing (MLST) database

A reference database with MLST alleles and a file which defines the sequence type profiles as a combination of alleles were downloaded from pubMLST on July 25, 2020, using the getmlst.py script included in the SRST2 github repository [49].

#### Gene detection and implementation of decision algorithm

The presence of specific genes in the genomes of bacterial strains was evaluated directly from raw reads using the mapping-based gene detection tool SRST2 v0.2.0 [49]. Reference databases described above and read sets (fastQ files) were used as input. Briefly, the software package SRST2 aligns the reads to each of the reference alleles included in the database and computes alignment scores, percentage coverage breadth, coverage depth, divergence, and mismatches [40]. The maximum number of mismatches allowed during mapping was set at 50 instead of the default value of 10. A gene was considered present if a homologue was found with a divergence of maximum 15% (instead of the default value of 10%) and a minimum coverage of 90% towards one of the alleles from the reference database. Deviation from the default SRST2 parameter values was necessary to successfully detect marker, haemin biosynthesis, and capsule genes in a genetically diverse dataset of *Haemophilus* strains and was optimized to allow for accurate species/serotype identification. In-house developed bash scripts were used to apply SRST2 v0.2.0 [49] on multiple datasets in parallel (to reduce calculation time) and to output additional text files reporting the detected (sub)species and serotype (based on the decision algorithm developed in this study) as well as annotation files to plot the presence of genes/species/serotype on a phylogenetic tree visualized with iTOL v6.1.2 [50]. All scripts are freely available at github (https://github.com/ngs-fzb/HaemoSeq) [45]. The scripts were run on an Ubuntu 12.04 server with Intel® Xeon® processor E5-2650 v4 @ 2.2 GHz and 48 GB of RAM available.

#### In silico serotyping

In silico serotyping was performed in two ways: (i) starting from raw sequencing reads using SRST2 and the decision algorithm developed in this study (see previous section) (script available at github under project name HaemoSeq, https://github.com/ngs-fzb/HaemoSeq [45]) and (ii) starting from assemblies using Hicap v1.0.3 with default values. Hicap was developed specifically for *H. influenzae* strains and does not evaluate region II genes from other *Haemophilus* spp. [46].

## Results

### Differentiation between *H. influenzae* and *H. haemolyticus* strains

Misclassifications of *H. haemolyticus* strains as *H. influenzae* are clinically the most relevant [3, 21–24]. Therefore, we first employed a pangenome-wide association study (panGWAS) using all (*n*=61) publicly available *H. haemolyticus* genome assemblies and all (*n*=68) publicly available fully closed *H. influenzae* genomes (Additional file 1: Table S1 and Additional file 2: Table S2), to identify new marker genes that specifically discriminate between these two species. Eighteen genes were present in all *H. influenzae* genomes and absent from all *H. haemolyticus* genomes while 43 genes were present in all *H. haemolyticus* genomes but in none of the *H. influenzae* genomes (Additional file 5: Table S5).

Classification based on only one marker gene has proven to be unsuccessful to distinguish *H. influenzae* from *H. haemolyticus* [21–23, 51, 52]. However, to reduce complexity and computation time, we chose only five out of 18 marker genes for *H. influenzae* and five out of 43 marker genes for *H. haemolyticus* as taxonomic classifiers for our new classification database (Table 1). The selection criteria were (i) genes with a sequence length as short as possible, but above 500 bp, (ii) genes with a fixed sequence length across strains of the training dataset, and (iii) only one gene per genomic cluster/operon or metabolic pathway to minimize the impact of large deletions/insertions (Additional file 6: Fig. S1).

**Table 1:** Selected *H. influenzae* and *H. haemolyticus* marker genes

Gene name	Marker for	Average size (bp)	Annotation (protein product)
*dat*	*H. influenzae*	1365	Diaminobutyrate--2-oxoglutarate aminotransferase
*hxuB*	*H. influenzae*	1659	Heme/hemopexin transporter protein
*oppC*	*H. influenzae*	936	Oligopeptide transport system permease protein
*pdxT*	*H. influenzae*	579	Pyridoxal 5'-phosphate synthase subunit
*phoB*	*H. influenzae*	696	Phosphate regulon transcriptional regulatory protein
*fklB*	*H. haemolyticus*	624	FKBP-type 22 kDa peptidyl-prolyl cis-trans isomerase
*hypD*	*H. haemolyticus*	1113	Hydrogenase maturation factor
*ndhI (nuoI)*	*H. haemolyticus*	576	NAD(P)H-quinone oxidoreductase subunit I
*resA*	*H. haemolyticus*	483	Thiol-disulfide oxidoreductase
*tpd*	*H. haemolyticus*	522	34 kDa membrane antigen

**Fig. 1:**
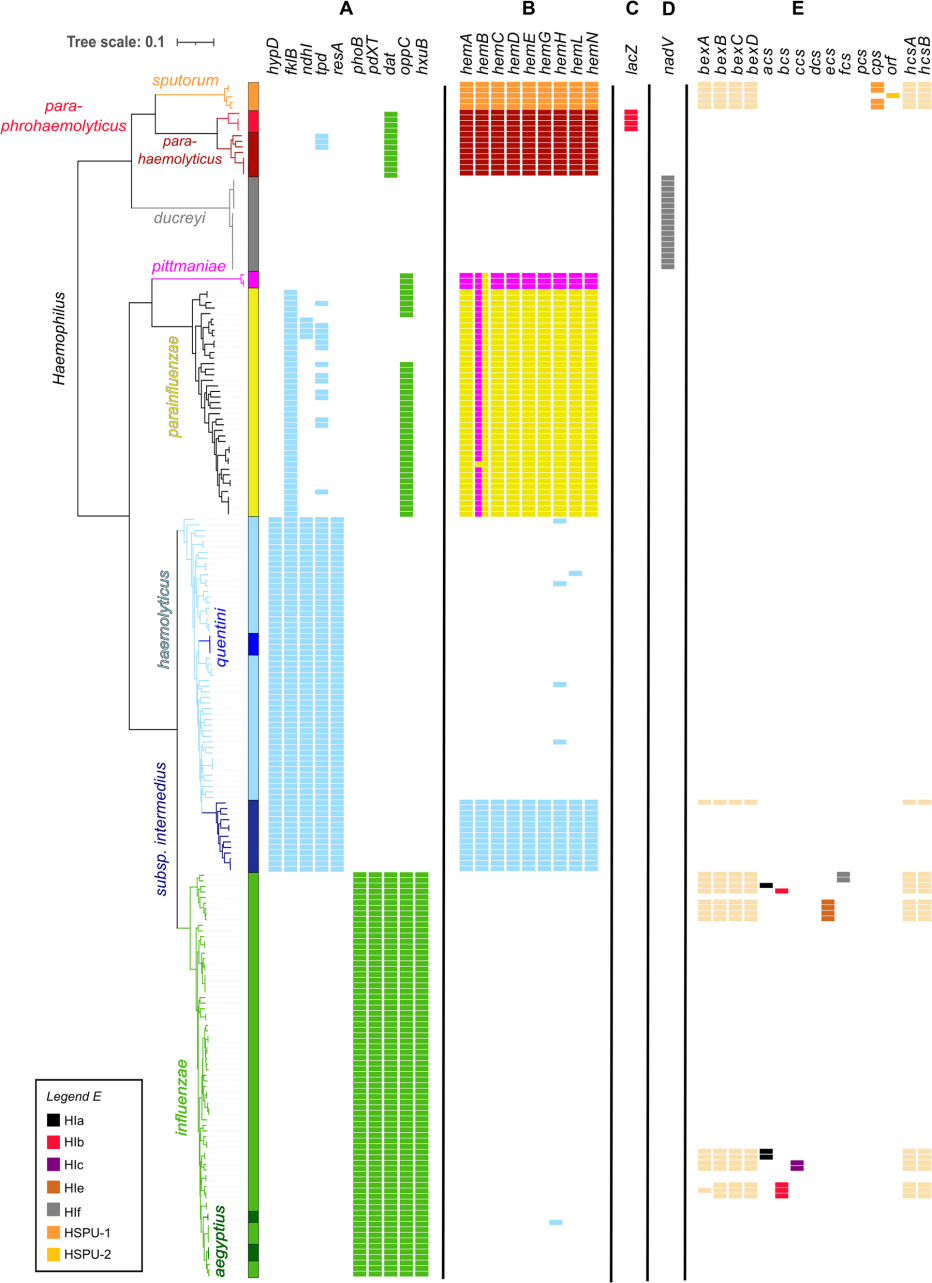
Presence and absence of marker genes in the training dataset. The phylogenetic tree is based on the alignment of 455 core genes (present in at least 90% of the strains) inferred from 215 whole genome sequencing datasets of human-related *Haemophilus* spp. **A** Presence/absence of marker genes that specifically discriminate between *H. haemolyticus* and *H. influenzae*. **B** Presence/Absence of haemin biosynthesis genes (hem*), which are colored according to the species identity of the reference alleles for which a valid hit was found. **C** Presence/absence of lacZ, a β-galactosidase gene that differentiates between *H. parahaemolyticus* and *H. paraphrohaemolyticus*. **D** Presence/absence of nadV, which is related to the *H. ducreyi* characteristic V factor independency. **E** Presence/absence of Region I (bex*), region II and region III (hcs*) capsule loci (in silico serotyping). All annotated *Haemophilus* spp. clades were separated with a strong local support value (100%)

Next, we extracted the corresponding DNA sequence (“reference allele”) of these ten genes from a *H. influenzae* and *H. haemolyticus* genome (Additional file 4: Table S4) and combined them into a SRST2 compatible reference database [40]. The ability to detect these genes direct from raw reads was evaluated using simulated read datasets from our training dataset (Fig. 1). We considered genes as “present” if a sequence was detected with a nucleotide identity of at least 85% (corresponding to a maximum divergence of 15%) and a coverage breadth of at least 90% in comparison to a reference allele. As a result, severely truncated genes or highly diverged homologs are regarded as “absent.”

All five *H. haemolyticus* marker genes were identified with SRST2 among all the *H. haemolyticus* strains including phylogenetically related strains previously termed *H. quentini*, and no hits were detected among *H. influenzae* strains (Fig. 1A). Likewise, all five *H. influenzae* marker genes were identified in all *H. influenzae* strains, including closely related *H. aegyptius* strains, while there were no hits among *H. haemolyticus* strains. Several *H. haemolyticus* and *H. influenzae* marker genes were also detected in other *Haemophilus* spp. but not more than three per strain (Fig. 1A).

### Differentiation of other *Haemophilus* (sub)species

The limited number of publicly available WGS data for *Haemophilus* spp. strains other than *H. haemolyticus* and *H. influenzae* discouraged the use of a panGWAS approach to find additional species-specific marker genes. Instead, we added a reference allele of the haemin biosynthesis loci from a H. parainfluenzae (hem*-HpI), H. parahaemolyticus (hem*-HpH), *H. sputorum* (hem*-HS), *H. pittmaniae* (hem*-HP), and *H. haemolyticus* (hem*-HH) genome to our classification database (Additional file 4: Table S4).

Although multiple *Haemophilus* spp. possess the haemin loci, we could restrict the valid hits (i.e., the alleles considered as present) exclusively to the reference alleles from the species the strain belonged to by employing conservative gene coverage and nucleotide identity thresholds (sequence divergence <15% and sequence coverage breadth ≥90%) (Fig. 1B). An exception was *hemB*, *which* did not show enough sequence divergence between *H. parainfluenzae* and *H. pittmaniae* strains, leading to the detection of both reference alleles (*hemB-HP* and *hemB-HpI*) for strains from both species (Fig. 1B). In addition, close homologs to the *H. parahaemolyticus* haemin reference genes (hem*-HpH) were also found in all *H. paraphrohaemolyticus* strains (Fig. 1B).

Haemin loci were only detected in *H. haemolyticus* subsp. *intermedius* strains that were reported to be haemin independent [53] (Fig. 1B and Additional file 2: Table S2).

To be able to differentiate between *H. paraphrohaemolyticus* (β-galactosidase positive) and *H. parahaemolyticus* (β-galactosidase negative), we added a β-galactosidase reference gene (*lacZ*) from a *H. paraphrohaemolyticus* strain to our classification database (Fig. 1C and Additional file 4: Table S4).

Finally, as *Haemophilus ducreyi* strains are lacking haemin biosynthesis genes, we included the gene *nadV* in our classification database, which is related to the *H. ducreyi* characteristic of V factor independency [3]. We exclusively found the *nadV* allele in *H. ducreyi* genomes from our training dataset and in none of the other *Haemophilus* spp. (Fig. 1D).

### Detection of capsule loci

We then evaluated whether we could detect capsule loci direct from raw sequence data in order to determine the serotype of *Haemophilus* spp. strains. To this end, we added reference alleles of region I (*bexA/B/C/D*), region II (determines the serotype), and region III (*hcsA/B*) of three *Haemophilus* spp. to our classification database (see the “Methods” section and Additional file 4: Table S4). In the training dataset, the predicted presence/absence of capsule loci was in agreement with the reported serotypes (available from literature for *H. influenzae* and *H. sputorum*) and with the assembly-based in silico predictions from Hicap (limited to *H. influenzae* strains) (Fig. 1E and Additional file 2: Table S2).

### Implementation of a decision algorithm for species classification and serotyping

**Fig. 2:**
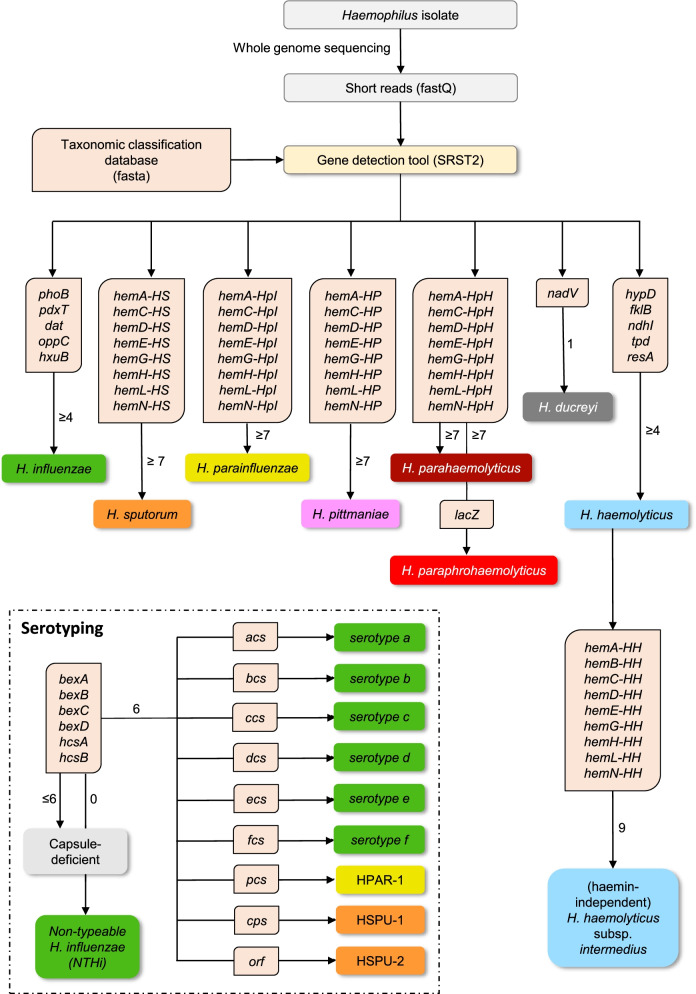
Decision algorithm to classify human-related strains of *Haemophilus* spp. based on whole genome sequencing data. The number next to the arrow specifies the minimum number of marker genes that needs to be detected before a (sub)species tag is attributed to the strain

Based on the analysis of the training dataset, we concluded the design of the taxonomic classification database, finally comprising 36 marker genes (Fig. 2). Next, we developed a gene presence/absence-based decision algorithm and combined it with the gene detection algorithm in a simple bash script. According to our decision algorithm, strains are identified as *H. influenzae* or *H. haemolyticus* when at least four out of five marker genes of the respective species are detected. We allowed one missing gene to compensate for potential gene loss events or a sequencing artifact. Likewise, the presence of at least seven out of eight haemin biosynthesis alleles (excluding *hemB*) are required to classify a strain as *H. pittmaniae*, *H. sputorum*, *H. parainfluenzae*, or *H. para(phro)haemolyticus*. *H. paraphrohaemolyticus* is distinguished from *H. parahaemolyticus* by the presence of a *lacZ* gene. When all capsule loci are detected, the serotype is determined based on the presence of specific region II alleles. Absence of at least one capsule gene is translated into a capsule-deficient strain and absence of all capsule genes in *H. influenzae* strains defines non-typeable *H. influenzae* (NTHi). Finally, presence of all nine haemin biosynthesis genes in the genome of a *H. haemolyticus* strain is indicative of a haemin-independent phenotype related to *H. haemolyticus* subsp. *intermedius*. The detection of (unexpected) marker genes from different *Haemophilus* species could further point towards mixed cultures or a recombination event, but our classification approach cannot rule out mixed populations of two strains from the same *Haemophilus* sp. or contamination with non-*Haemophilus* spp. strains.

Starting from the simulated read datasets of the training dataset (*n*=215), our new script produced classification and serotyping results in less than 15 min using 30 CPU threads (on average 1.7 min per read dataset).

### Validation of the new in silico classification/serotyping algorithm

Next, the classification algorithm was employed to classify 1329 publicly available WGS datasets of *Haemophilus* spp. strains (evaluation dataset). Predicted species classifications were in agreement with the SRA reported taxonomy for 1324/1329 (99.6%) datasets. Five reported *H. influenzae* strains were reclassified by our classification algorithm as *H. parainfluenzae* (*n*=4) and *H. haemolyticus* subsp. *intermedius* (*n*=1) (Additional file 2: Table S2), which was confirmed by a core gene alignment-based phylogenetic analysis of these strains together with our training dataset strains (Additional file 7: Fig. S2). All of the reconfirmed *H. influenzae* strains harbored the full dataset of *H. influenzae* markers and two also had one additional *H. haemolyticus* marker (Additional file 8: Table S6). All five *H. haemolyticus* marker genes and none of the *H. influenzae* markers were found in the *H. haemolyticus* strains. All *H. parainfluenzae* strains harbored the *haemin* biosynthesis genes and a maximum of two *H. haemolyticus* or *H. influenza* markers were detected. Lastly, *nadV* was only detected in *H. ducreyi* datasets.

Capsule loci were found in 534/1055 *H. influenzae* and 12/144 *H. parainfluenzae* strains from the evaluation dataset (Additional file 2: Table S2 and additional file 8: Table S6). Our serotype prediction was confirmed for 369/371 *H. influenzae* with reported serotyping data available from SRA and for 11 previously serotyped *H. parainfluenzae* strains [25, 54], corresponding to an overall concordance of 99.5% (additional file 2: Table S2). Furthermore, all predicted serotypes of *H. influenzae* strains (*n*=521) were in agreement with assembly-based Hicap predictions (Additional file 2: Table S2). However, the presumed mixed populations (*n*=2) with multiple serotypes and capsule-deficient strains (*n*=11) predicted by our algorithm were not identified as such by Hicap.

### Retrospective reclassification of clinical *Haemophilus* spp. isolates classified as *H. influenzae* during routine diagnostics

**Fig. 3:**
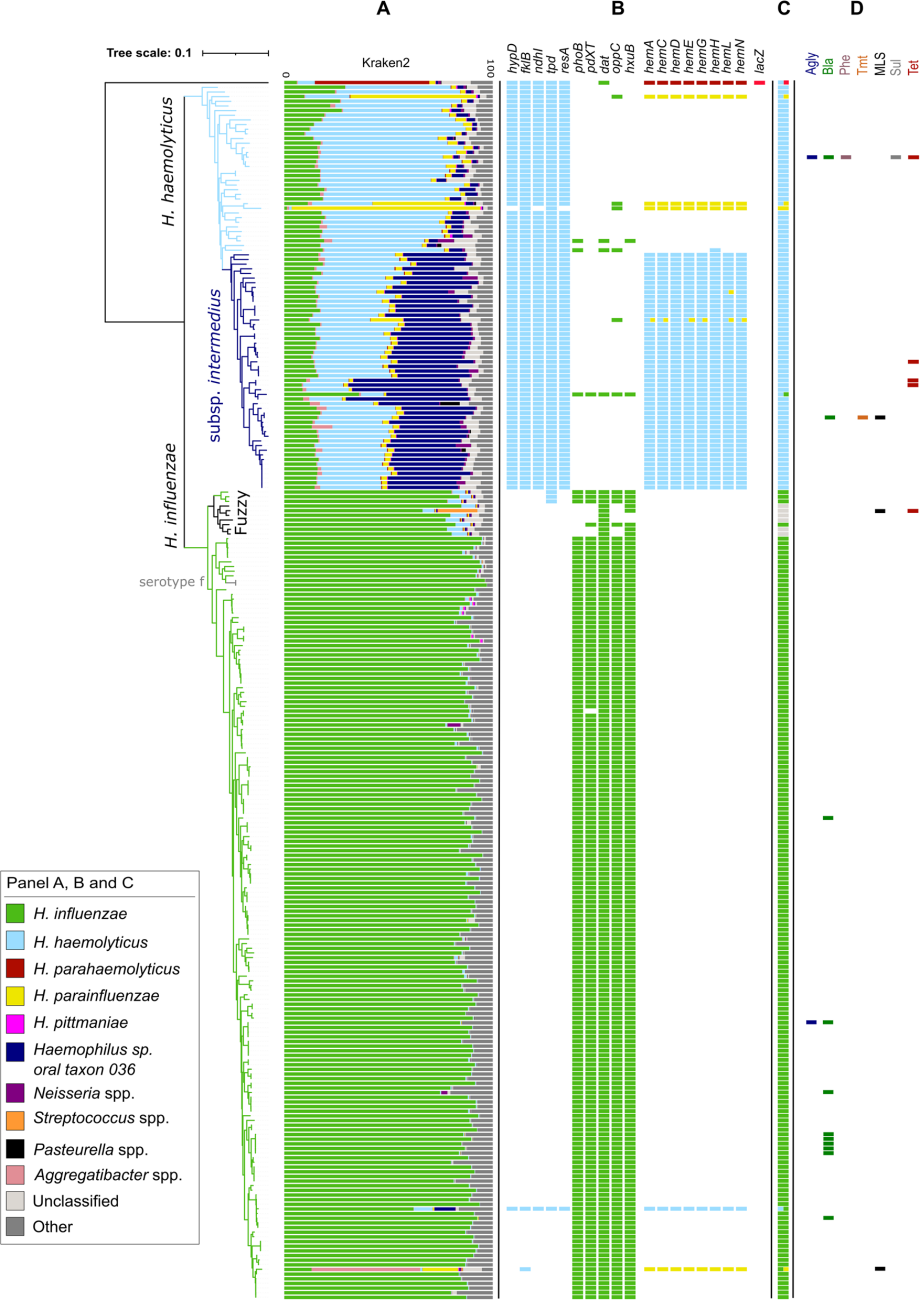
Phylogeny of 262 clinical *Haemophilus* spp. isolates from a German cohort. The phylogenetic tree is based on the alignment of 104 core genes (present in at least 90% of the strains). **A** Kraken2 read classification output. The length of a bar is proportional to the percentage of reads that are assigned to the respective taxon (as indicated by the color). One *H.* influenzae culture (located in the phylogenetic tree in the “fuzzy” clade) was likely contaminated with a *Streptococcus* sp. strain (19% of the reads assigned to this species) and another one with an *Aggregatibacter* sp. strain (52% reads assigned to this species). **B** Presence/absence of marker genes included in our new taxonomic classification database. **C** Final classification output of the decision algorithm. Mixed colors represent the presence of multiple full marker patterns, indicating multiple distinct *Haemophilus* species. **D** Presence/absence of antibiotic resistance genes included in a public resistance database. Color codes correlate to the antibiotic class to which the gene confers resistance: aminoglycosides (Agly), β-lactam antibiotics (Bla), phenicols (Phe), trimethoprim (Tmt), macrolide-lincosamide-streptogramin (MLS), sulfonamides (Sul), and tetracyclines (Tet)

In total, 262 clinical *Haemophilus* isolates were collected from 250 patients in three Northern German hospitals between 2008 and 2013 and were at that time classified by MALDI-TOF MS as *H. influenzae* (Additional file 3: Table S3). The isolates were also recultivated and sequenced on an Illumina platform. First, we classified the resulting sequencing reads of all 262 presumptive *H. influenzae* strains with kraken2, another popular read-based taxonomic classification tool [31]. However, kraken2 classified only 173 out of 262 isolates unambiguously as *H. influenzae* (Fig. 3A). The other isolates showed a mixed species profile with reads assigned to multiple *Haemophilus* spp., mainly *H. haemolyticus*, *H. influenzae,* and *Haemophilus* sp. oral taxon 036, making it difficult to unambiguously assign a taxonomic label to the strains (Fig. 3A).

We then applied our classification algorithm on the sequencing data of all 262 *Haemophilus* isolates. In total, 166 out of 262 (63.4%) datasets were unambiguously classified by our decision algorithm as *H. influenzae*, two of them belonging to serotype f (Fig. 3B/C and Additional file 3: Table S3). Six strains could not be classified by our algorithm due to the absence of a complete species-specific marker pattern nor could they be typed by multi-locus sequence typing (MLST) due to a missing *fucK* gene (Fig. 3B/C, Additional file 3: Table S3 and Additional file 9: Table S7). Together with three *H. influenzae* strains that harbored one *H. haemolyticus* marker, they formed a separate clade in our phylogenetic tree, which we labeled “fuzzy” due to the aberrant marker patterns and kraken2 profile (Fig. 3A/B). One of these fuzzy strains was reclassified with current MALDI-TOF MS equipment/software as *H. haemolyticus*, while one showed high MALDI scores for both *H. haemolyticus/H. influenzae* (Additional file 3: Table S3).

Remarkably, 83 out of 262 (31.7%) presumptive *H. influenzae* isolates were unambiguously classified as *H. haemolyticus* strains by our classification algorithm despite mixed kraken2 profiles (Fig. 3 A/B/C and Additional file 3: Table S3). Except for three isolates, marker genes of other *Haemophilus* spp. were not detected in these strains (Fig. 3B). The genomes of more than half of the *H. haemolyticus* strains (*n*=51) contained all genes involved in the biosynthesis of haemin and were therefore classified as *H. haemolyticus* subsp. *intermedius* strains by our algorithm (Fig. 3B and Additional file 3: Table S3). As the presence of genes in a strain’s genome does not guarantee that the corresponding proteins are expressed/functional, we also retrospectively evaluated the in vitro haemin independence for 44 *H. haemolyticus* strains which could be recultivated. Three of these 44 (6.8%) *H. haemolyticus* strains were still misclassified as *H. influenzae* with current MALDI-TOF MS equipment/software. The X-factor/haemin-independent phenotype was confirmed for 24 out of 25 of the *H. haemolyticus* subsp. *intermedius* strains while the haemin-dependent phenotype was confirmed for the other 19 *H. haemolyticus* strains (Additional file 3: Table S3).

For six clinical isolates, a full marker pattern of two different *Haemophilus* spp. was found by our classification algorithm, pointing towards a mixed population or laboratory contamination (Fig. 3B/C and Additional file 3: Table S3). The presence of multiple strains in the corresponding cultures was further strengthened by the unusually long (and fragmented) WGS assemblies (>2.5 Mbp) (Additional file 3: Table S3). In addition, one isolate was unambiguously classified as *H. parainfluenzae*, which also agreed with the kraken2 classification.

Based on the corresponding metadata we found that 31 out of 140 (24.3%) lower respiratory tract isolates were *H. haemolyticus* strains, which is highly unexpected (Additional file 3: Table S3). Moreover, 16 of these strains were cultured from patients with suspected and/or proven pneumoniae, and for 11 of these patients (69%) no other relevant pneumonia-related pathogen was found. In addition, *H. haemolyticus* strains were the only plausible causative agent in four out of nine (44.4%) patients with mouth abscesses as no other pathogen was detected (Additional file 3: Table S3). This points towards an unexpected pathogenic role of *H. haemolyticus* at least in these two clinical conditions.

Finally, we also determined the exogeneously acquired resistome using the same gene detection tool, combined with a public antibiotic resistance database. One or more resistance genes were found in 16 out of 262 datasets, including 9 *H. influenzae*, *5 H. haemolyticus*, and 2 putative mixed isolates (Fig. 3D and Additional file 3). All nine *H. influenzae* strains and one *H. haemolyticus* strain carried the common *bla*_TEM-1D_ gene associated with ampicillin resistance. The tetracycline resistance-associated gene *tetB* was identified in four *H. haemolyticus* strains (Fig. 3D and Additional file 3). Retrospective pDST to confirm the prediction of antimicrobial resistance was possible for 10/16 isolates (Additional file 10: Table S8). Of those, all *bla*_TEM-1D_ (*n*=9)*,* and all *tetB* (*n*=3) positive isolates were confirmed to be phenotypically resistant to ampicillin (but not ceftriaxone) and tetracycline, respectively. One *H. haemolyticus* strain was predicted ampicillin resistant based on the presence of the *bla*_*Z*_ gene, but this was not confirmed by pDST (false positive). In addition, one *H. influenzae* strain with a *bla*_TEM-1D_ gene, which codes for a β-lactamase that is typically inhibited by sulbactam [55], was phenotypically resistant to ampicillin/sulbactam. This indicates an additional mode of resistance that cannot be explained by a gene presence/absence approach (false negative) (Additional file 10: Table S8).

## Discussion

Correct species identification and antibiotic resistance prediction are crucial for proper diagnosis and antimicrobial stewardship. However, differentiation between some *Haemophilus* spp. has proven difficult with current diagnostics. Here, we present a taxonomic marker database and WGS-based classification/serotyping algorithm for human-related *Haemophilus* spp., which can easily be combined with the detection of acquired resistance genes.

The newly developed lightweight taxonomic database (only 110 kilobytes) comprises 36 genes, including newly identified *Haemophilus* sp. marker genes as well as genes that are involved in important metabolic pathways (haemin biosynthesis) and virulence mechanisms (capsule loci). The classification algorithm is based on the detection of these marker genes directly from short reads (fastQ files) using SRST2 and allows to differentiate between *H. influenzae*, *H. haemolyticus*, *H. parahaemolyticus*, *H. paraphrohaemolyticus*, *H. parainfluenzae*, *H. sputorum*, *H. ducreyi*, and *H. pittmaniae*. *H. quentini* and *H. aegyptius* are not recognized as separate species but classified as *H. haemolyticus* and *H. influenzae*, respectively.

The inclusion of capsule loci in our classification database allows to predict the serotype of *Haemophilus spp*. strains. We considered *H. influenzae* strains as NTHi when none of the capsule loci were detected. When at least one capsule loci was found, but not all, we labeled the strains as “capsule-deficient.” The genetics of these mutants have mainly been investigated for *H. influenzae* serotype b [56]. In these cases, deletion of *bexA* has been identified as the cause for the capsule loss. Consequences of other deletions would need further experimental investigation. Moreover, the in silico detection of capsule genes does not guarantee that a capsule is formed as gene expression can be affected by many other factors such as the presence of specific mutations/indels, the action of regulatory proteins, or the microenvironment [57, 58]. Similarly, the presence of haemin genes allows for subspeciation of *Haemophilus* strains, but it is not a warranty for a fully functioning haemin biosynthesis pathway.

Our classification method showed high concordance with the reported serotype (99.5%) and species classification (99.6%) for the evaluation dataset (*n*=1329). Based on our results, we propose that some presumed *H. influenzae* strains available at SRA should be reclassified as *H. parainfluenzae* and *H. haemolyticus*, respectively. However, more sequencing data for less prevalent *Haemophilus* spp. such as *H. para(phro)haemolyticus*, *H. sputorum*, *H. ducreyi*, and *H. pittmaniae* are needed to further validate the robustness of our classification algorithm.

Next to publicly available data, we also analyzed a new dataset of 262 *Haemophilus* spp. isolates from 250 patients in Germany that were all classified as *H. influenzae* by MALDI-TOF MS at the time of diagnosis (between 2008 and 2013). In this cohort, our algorithm reclassified almost one third of the *H. influenzae* strains as *H. haemolyticus*, also pointing towards a role of *H. haemolyticus* as human pathogen. Few strains could not be classified by our decision algorithm due to an aberrant marker pattern. These strains remind of previously described “fuzzy” species [59, 60] and were most closely related to *H. influenzae* strains. The high *H. haemolyticus* misclassification rate could be explained by an incomplete MALDI-TOF mass spectrum database that was used at the time of diagnosis [61, 62]. Nevertheless, the high prevalence of *H. haemolyticus* strains in our strain dataset is in clear contrast to findings of a similar study [63], in which only a few *H. haemolyticus* strains were found among clinical isolates obtained from patients in Germany. Another interesting finding was the presence of *H. haemolyticus* in aspirates of mouth abscesses. *H. influenzae* has been isolated from peritonsillar abscesses before, but *H. haemolyticus* has not been linked with this condition to the best of our knowledge [64]. The fact that a considerable number of the *H. haemolyticus* strains in this study were isolated from lower respiratory tract (LRT) specimens from patients with suspected or proven pneumonia could point towards another unexpected pathogenic role of *H. haemolyticus* in LRT infections. However, as LRT samples can also be contaminated with the bacterial flora of the upper respiratory tract [65] and humans can be colonized by multiple strains at the same time [4, 66, 67], further research is required to evaluate the pathogenic relevance of *H. haemolyticu*s.

WGS-based resistance prediction is increasingly being used to monitor antimicrobial resistance, which has become a major global public health threat, as well as for personalized therapy [68, 69]. However, resistance phenotype-genotype association studies for *Haemophilus* species are currently scarce. To determine the horizontally acquired resistome, we used the same gene detection tool (SRST2) that was used to determine the presence of our classification markers, in combination with a public resistance database [40, 70]. In the German cohort, we detected at least one resistance gene in 16 out of 262 datasets. Ampicillin resistance, associated with the presence of the *bla*_TEM-1D_ gene, and tetracycline resistance, associated with the presence of the *tetB* gene, could be confirmed in vitro by routine pDST. The identification of *Haemophilus* spp. strains with predicted resistance to multiple antibiotics (including β-lactam antibiotics) highlight the need for close monitoring of resistance evolution and spread of resistant strains in the future. The detection of resistance genes is, however, limited to rule-in antimicrobial resistance, and the absence of resistance genes in a specimen does not imply antimicrobial susceptibility. A follow-up study is envisaged which complements the gene detection approach with a mutation database and comprehensive pDSTs to determine sensitivity and specificity of a molecular antimicrobial resistance prediction for clinical *Haemophilus* spp. strains*.*

Overall, our WGS-based classification algorithm offers several advantages compared to other identification tools/techniques: (i) it can be used for species classification of human-related *Haemophilus* spp. as well as for in silico serotyping and prediction of haemin independency (ii), it is very fast and can be run on a basic laptop due to the lightweight classification database and low memory requirements, (iii) it outputs easy interpretable text files with gene presence/absence results and classification tags as well as iTol annotation files for visualization of the results on phylogenetic trees, and (iv) it can easily be combined with MLST typing and the detection of acquired resistance genes. On the other hand, the algorithm cannot detect species belonging to genera other than *Haemophilus* and only works well with short read data.

At this stage, we believe that WGS-based classification and especially resistance prediction should be regarded as a tool that complements other routine diagnostic methods. An additional algorithm that detects chromosomal resistance-related mutations as well as more phenotype-genotype studies and larger sample sizes will be necessary to further evaluate the use of WGS as a diagnostic method for *Haemophilus* spp. in clinical microbiology.

## Conclusions

We developed a lightweight gene database and fast assembly-free classification algorithm that is customized to identify clinically relevant *Haemophilus* spp. direct from raw short read WGS data. In addition, the algorithm can also determine the serotype of typeable *Haemophilus* strains and identify putative haemin-independent *H. haemolyticus* strains. Together with drug resistance prediction, this opens the door for improved diagnosis, surveillance, and treatment of *Haemophilus* spp. infections.

## Supplementary Information

**Additional file 1: Table S1.** Summary of the number of short read datasets used for each species in the training set, evaluation set, and German cohort.

**Additional file 2: Table S2.** Metadata and classification results for all samples from the training and evaluation set.

**Additional file 3: Table S3.** Metadata and classification results for all samples from German cohort.

**Additional file 4: Table S4.** Summary of all genes that were evaluated as taxonomic markers.

**Additional file 5: Table S5.** Summary of all *H. influenzae* and *H. haemolyticus* marker candidates that were identified using a panGWAS analysis.

**Additional file 6: Figure S1.** Genomic arrangement of selected marker genes.

**Additional file 7: Figure S2.** Phylogenetic tree comprising training set strains and misclassified strains from SRA.

**Additional file 8: Table S6.** SRST2 output for short read datasets from the evaluation set using the newly developed classification database.

**Additional file 9: Table S7.** SRST2 output for Illumina sequencing datasets from the German cohort using a public MLST database.

**Additional file 10: Table S8.** phenotypic drug susceptibility testing (pDST) results for isolates from the German cohort.

## Data Availability

The whole genome sequencing data generated in this study (Additional file 3: Table S3) have been submitted to the NCBI BioProject database [71] under accession number PRJEB43356 (https://www.ncbi.nlm.nih.gov/sra/?term=PRJEB43356) [72]. All other WGS datasets were downloaded from public repositories (NCBI and SRA), and accession numbers are available in Additional file 2: Table S2. All scripts, a manual to run these bash scripts, and the taxonomic classification database developed for this study are available at github under project name HaemoSeq, https://github.com/ngs-fzb/HaemoSeq [45]. The datasets supporting the conclusions of this article are included within the article (and its additional files).

## Abbreviations

LRT: Lower respiratory tract
MALDI-TOF MS: Matrix-assisted laser desorption ionization–time of flight mass spectrometry
MIC: Minimum inhibitory concentration
MLST: Multi-locus sequencing typing
NCBI: National Center for Biotechnology Information
NTHi: Non-typeable *H. influenzae*
panGWAS: Pangenome-wide association study
pDST: Phenotypic drug susceptibility testing
PCR: Polymerase chain reaction
SRA: Sequence read archive
WGS: Whole genome sequencing

## References

[1] Winslow C, Broadhurst J, Buchanan R, Krumwiede C, Rogers L, Smith G (1917). The Families and Genera of the Bacteria: Preliminary Report of the Committee of the Society of American Bacteriologists on Characterization and Classification of Bacterial Types. J Bacteriol..

[2] Thjötta T, Avery OT (1921). Studies on bacterial nutrition : II. Growth accessory substances in the cultivation of Hemophilic bacilli. J Exp Med.

[3] Nørskov-Lauritsen N (2014). Classification, identification, and clinical significance of Haemophilus and Aggregatibacter species with host specificity for humans. Clin Microbiol Rev..

[4] Mukundan D, Ecevit Z, Patel M, Marrs CF, Gilsdorf JR (2007). Pharyngeal colonization dynamics of Haemophilus influenzae and Haemophilus haemolyticus in healthy adult carriers. J Clin Microbiol..

[5] Van Eldere J, Slack MPE, Ladhani S, Cripps AW (2014). Non-typeable Haemophilus influenzae, an under-recognised pathogen. Lancet Infect Dis..

[6] Peltola H (2000). Worldwide Haemophilus influenzae type b disease at the beginning of the 21st century: global analysis of the disease burden 25 years after the use of the polysaccharide vaccine and a decade after the advent of conjugates. Clin Microbiol Rev.

[7] McCormick DW, Molyneux EM (2011). Bacterial meningitis and haemophilus influenzae type b conjugate Vaccine, Malawi. Emerg Infect Dis..

[8] Musher DM. Haemophilus species. In Medical Microbiology: 4th edition (Samuel Baron). The University of Texas Medical Branch at Galveston. Medical Microbiology. University of Texas Medical Branch at Galveston; 1996.

[9] Bakaletz LO, Novotny LA (2018). Nontypeable Haemophilus influenzae (NTHi). Trends Microbiol.

[10] WHO priority list [Internet]. Available from: https://www.who.int/medicines/areas/rational_use/prioritization-of-pathogens/en/.

[11] Simberkoff MS, Goldman L, Schafer AI (2012). Haemophilus and Moraxella infections. Goldman’s Cecil Medicine: 24th edition.

[12] Anderson R, Wang X, Briere EC, Katz LS, Cohn AC, Clark TA (2012). Haemophilus haemolyticus isolates causing clinical disease. J Clin Microbiol..

[13] Nørskov-Lauritsen N, Bruun B, Andersen C, Kilian M (2012). Identification of haemolytic Haemophilus species isolated from human clinical specimens and description of Haemophilus sputorum sp. nov. Int J Med Microbiol..

[14] Nørskov-Lauritsen N, Bruun B, Kilian M (2005). Multilocus sequence phylogenetic study of the genus Haemophilus with description of Haemophilus pittmaniae sp. nov. Int J Syst Evol Microbiol..

[15] Kus JV, Shuel M, Soares D, Hoang W, Law D, Tsang RSW. Identification and characterization of “Haemophilus quentini” strains causing invasive disease in Ontario, Canada (2016 to 2018). J Clin Microbiol. 2019;57(12):e01254-19. 10.1128/JCM.01254-19 PMC6879273 31578259

[16] Murphy TF, Kirkham C, Sikkema DJ (1992). Neonatal, urogenital isolates of biotype 4 nontypeable Haemophilus influenzae express a variant P6 outer membrane protein molecule. Infect Immun..

[17] Brenner DJ, Mayer LW, Carlone GM, Harrison LH, Bibb WF, Brandileone MC (1988). Biochemical, genetic, and epidemiologic characterization of Haemophilus influenzae biogroup aegyptius (Haemophilus aegyptius) strains associated with Brazilian purpuric fever. J Clin Microbiol..

[18] Lewis DA, Mitjà O (2016). Haemophilus ducreyi: from sexually transmitted infection to skin ulcer pathogen. Curr Opin Infect Dis.

[19] Cooke FJ, Slack MPE. Infectious diseases. In: Fourth. 2017.

[20] Hedegaard J, Okkels H, Bruun B, Kilian M, Mortensen KK, Nørskov-Lauritsen N (2001). Phylogeny of the genus Haemophilus as determined by comparison of partial infB sequences. The GenBank accession numbers for the sequences reported in this paper are AJ289629 through AJ289694, AJ290742 through AJ290767, and AJ295746. Microbiology..

[21] Pickering J, Richmond PC, Kirkham L-AS (2014). Molecular tools for differentiation of non-typeable Haemophilus influenzae from Haemophilus haemolyticus. Front Microbiol..

[22] Osman KL, Jefferies JMC, Woelk CH, Devos N, Pascal TG, Mortier MC (2018). Patients with chronic obstructive pulmonary disease harbour a variation of Haemophilus species. Sci Rep..

[23] Price EP, Harris TM, Spargo J, Nosworthy E, Beissbarth J, Chang AB (2017). Simultaneous identification of Haemophilus influenzae and Haemophilus haemolyticus using real-time PCR. Future Microbiol..

[24] Nürnberg S, Claus H, Krone M, Vogel U, Lâm TT. Discriminative potential of the vitek MS in vitro diagnostic device regarding Haemophilus influenzae and Haemophilus haemolyticus. J Clin Microbiol Am Soc Microbiol. 2020;58:e00278-20. 10.1128/JCM.00278-20 PMC7448641 32404483

[25] Sierra Y, González-Díaz A, Carrera-Salinas A, Berbel D, Vázquez-Sánchez D, Tubau F (2021). Genome-wide analysis of urogenital and respiratory multidrug-resistant Haemophilus parainfluenzae. J Antimicrob Chemother..

[26] Kitts PA, Church DM, Thibaud-Nissen F, Choi J, Hem V, Sapojnikov V (2016). Assembly: a resource for assembled genomes at NCBI. Nucleic Acids Res..

[27] Escalona M, Rocha S, Posada D (2016). A comparison of tools for the simulation of genomic next-generation sequencing data. Nat Rev Genet.

[28] Leinonen R, Sugawara H, Shumway M (2011). The sequence read archive. Nucleic Acids Res.

[29] NCBI [Internet]. Available from: https://www.ncbi.nlm.nih.gov/.

[30] Andrews S. FastQC [Internet]. Available from: http://www.bioinformatics.babraham.ac.uk/projects/fastqc/.

[31] Wood DE, Lu J, Langmead B (2019). Improved metagenomic analysis with Kraken 2. Genome Biol..

[32] European Society of Clinical Microbiology and Infectious Diseases. EUCAST clinical breakpoints [Internet]. Available from: https://eucast.org/clinical_breakpoints/.

[33] Seemann T. Shovill [Internet]. Available from: https://github.com/tseemann/shovill.

[34] Bankevich A, Nurk S, Antipov D, Gurevich AA, Dvorkin M, Kulikov AS (2012). SPAdes: a new genome assembly algorithm and its applications to single-cell sequencing. J Comput Biol..

[35] Souvorov A, Agarwala R, Lipman DJ (2018). SKESA: strategic k-mer extension for scrupulous assemblies. Genome Biol.

[36] Petit RA. Shovill-se [Internet]. Available from: https://github.com/rpetit3/shovill/blob/single-end-reads/bin/shovill-se.

[37] Seemann T (2014). Prokka: rapid prokaryotic genome annotation. Bioinformatics..

[38] Page AJ, Cummins CA, Hunt M, Wong VK, Reuter S, Holden MTG (2015). Roary: rapid large-scale prokaryote pan genome analysis. Bioinformatics..

[39] Bayliss SC, Thorpe HA, Coyle NM, Sheppard SK, Feil EJ (2019). PIRATE: A fast and scalable pangenomics toolbox for clustering diverged orthologues in bacteria. Gigascience..

[40] Inouye M, Dashnow H, Raven LA, Schultz MB, Pope BJ, Tomita T (2014). SRST2: rapid genomic surveillance for public health and hospital microbiology labs. Genome Med..

[41] Price MN, Dehal PS, Arkin AP (2010). FastTree 2 - approximately maximum-likelihood trees for large alignments. PLoS One.

[42] Rambaut A. Figtree [Internet]. Available from: https://github.com/rambaut/figtree.

[43] Letunic I, Bork P (2019). Interactive Tree of Life (iTOL) v4: recent updates and new developments. Nucleic Acids Res..

[44] Inkscape [Internet]. Available from: https://inkscape.org/.

[45] Diricks M. HaemoSeq. 2021. Github. https://github.com/ngs-fzb/HaemoSeq.

[46] Watts SC, Holta KE. HICAP: In silico serotyping of the haemophilus influenzae capsule locus. J Clin Microbiol. 2019;57(6):e00190-19. 10.1128/JCM.00190-19 PMC6535587 30944197

[47] Potts CC, Topaz N, Rodriguez-Rivera LD, Hu F, Chang HY, Whaley MJ (2019). Genomic characterization of Haemophilus influenzae: a focus on the capsule locus. BMC Genomics..

[48] Pinto M, González-Díaz A, Machado MP, Duarte S, Vieira L, Carriço JA, et al. Insights into the population structure and pan-genome of Haemophilus influenzae. Infect Genet Evol. 2019;(67):126–35. 10.1016/j.meegid.2018.10.025 30391557

[49] SRST2 [Internet]. Available from: https://github.com/katholt/srst2.

[50] Letunic I, Bork P (2007). Interactive Tree Of Life (iTOL): an online tool for phylogenetic tree display and annotation. Bioinformatics..

[51] Connor TR, Corander J, Hanage WP (2012). Population subdivision and the detection of recombination in non-typable Haemophilus influenzae. Microbiology (United Kingdom)..

[52] Witherden EA, Bajanca-Lavado MP, Tristram SG, Nunes A (2014). Role of inter-species recombination of the ftsI gene in the dissemination of altered penicillin-binding-protein-3-mediated resistance in Haemophilus influenzae and Haemophilus haemolyticus. J Antimicrob Chemother..

[53] Harris TM, Price EP, Sarovich DS, Nørskov-Lauritsen N, Beissbarth J, Chang AB, et al. Comparative genomic analysis identifies x-factor (Haemin)-independent haemophilus haemolyticus: a formal re-classification of “haemophilus intermedius”. Microb Genomics. 2020;6(1):e000303. 10.1099/mgen.0.000303 PMC7067038 31860436

[54] González-Díaz A, Tubau F, Pinto M, Sierra Y, Cubero M, Càmara J, et al. Identification of polysaccharide capsules among extensively drug-resistant genitourinary Haemophilus parainfluenzae isolates. Sci Rep. 2019;9(1):4481. 10.1038/s41598-019-40812-2 PMC6418240 30872664

[55] Bush K, Jacoby GA (2010). Updated functional classification of β-lactamases. Antimicrob Agents Chemother..

[56] Satola SW, Collins JT, Napier R, Farley MM (2007). Capsule gene analysis of invasive haemophilus influenzae: accuracy of serotyping and prevalence of IS1016 among nontypeable isolates. J Clin Microbiol..

[57] Cope EK, Goldstein-Daruech N, Kofonow JM, Christensen L, McDermott B, Monroy F (2011). Regulation of virulence gene expression resulting from streptococcus pneumoniae and nontypeable haemophilus influenzae interactions in chronic disease. Miyaji EN, editor. PLoS One..

[58] Bervoets I, Charlier D (2019). Diversity, versatility and complexity of bacterial gene regulation mechanisms: opportunities and drawbacks for applications in synthetic biology. FEMS Microbiol Rev.

[59] De Gier C, Kirkham LAS, NØrskov-Lauritsen N. (2015). Complete deletion of the fucose operon in haemophilus influenzae is associated with a cluster in multilocus sequence analysis-based phylogenetic group II related to haemophilus haemolyticus: implications for identification and typing. J Clin Microbiol..

[60] Price EP, Sarovich DS, Nosworthy E, Beissbarth J, Marsh RL, Pickering J (2015). Haemophilus influenzae: using comparative genomics to accurately identify a highly recombinogenic human pathogen. BMC Genomics..

[61] Frickmann H, Christner M, Donat M, Berger A, Essig A, Podbielski A, et al. Rapid Discrimination of Haemophilus influenzae, H. parainfluenzae, and H. haemolyticus by Fluorescence In Situ Hybridization (FISH) and Two Matrix-Assisted Laser-Desorption-Ionization Time-of-Flight Mass Spectrometry (MALDI-TOF-MS) Platforms. PLoS One. 2013;8(4):e63222. 10.1371/journal.pone.0063222 PMC3639997 23646201

[62] Zhu B, Xiao D, Zhang H, Zhang Y, Gao Y, Xu L, et al. MALDI-TOF MS distinctly differentiates nontypable Haemophilus influenzae from Haemophilus haemolyticus. PLoS One. 2013;8(2):e56139. 10.1371/journal.pone.0056139 PMC3573053 23457514

[63] Frickmann H, Podbielski A, Essig A, Schwarz NG, Zautner AE (2014). Difficulties in species identification within the genus Haemophilus—a pilot study addressing a significant problem for routine diagnostics. Eur J Microbiol Immunol..

[64] Slouka D, Hanakova J, Kostlivy T, Skopek P, Kubec V, Babuska V (2020). Epidemiological and microbiological aspects of the peritonsillar abscess. Int J Environ Res Public Health..

[65] Loens K, Van Heirstraeten L, Malhotra-Kumar S, Goossens H, Ieven M (2009). Optimal sampling sites and methods for detection of pathogen possibly causing community-acquired lower respiratory tract infections. J Clin Microbiol.

[66] Murphy TF, Sethi S, Klingman KL, Brueggemann AB, Doern GV (1999). Simultaneous respiratory tract colonization by multiple strains of nontypeable Haemophilus influenzae in chronic obstructive pulmonary disease: implications for antibiotic therapy. J Infect Dis..

[67] Smith-Vaughan HC, Leach AJ, Shelby-James TM, Kemp K, Kemp DJ, Mathews JD (1996). Carriage of multiple ribotypes of non-encapsulated Haemophilus influenzae in Aboriginal infants with otitis media. Epidemiol Infect..

[68] Gröschel MI, Walker TM, van der Werf TS, Lange C, Niemann S, Merker M (2018). Pathogen-based precision medicine for drug-resistant tuberculosis. Leong JM, editor. PLOS Pathog.

[69] Hendriksen RS, Bortolaia V, Tate H, Tyson GH, Aarestrup FM, McDermott PF (2019). Using genomics to track global antimicrobial resistance. Front Public Health.

[70] Gupta SK, Padmanabhan BR, Diene SM, Lopez-Rojas R, Kempf M, Landraud L (2014). ARG-annot, a new bioinformatic tool to discover antibiotic resistance genes in bacterial genomes. Antimicrob Agents Chemother..

[71] Bioproject database [Internet]. Available from: https://www.ncbi.nlm.nih.gov/bioproject/.

[72] Diricks M, Kohl TA, Käding N, Leshchinskiy V, Hauswaldt S, Jiménez Vázquez O, et al. Whole genome sequencing based classification of human-related Haemophilus species and detection of antimicrobial resistance genes. BioProject PRJEB43356, NCBI Sequence Read Archive 2021. https://www.ncbi.nlm.nih.gov/sra/?term=PRJEB43356 (2021). 10.1186/s13073-022-01017-x PMC8830169 35139905

