# Correction to: Whole genome sequencing-based classifcation of human-related Haemophilus species and detection of antimicrobial resistance genes

**DOI:** 10.1186/s13073-022-01028-8

**Published:** 2022-02-24

**Authors:** Margo Diricks, Thomas A. Kohl, Nadja Käding, Vladislav Leshchinskiy, Susanne Hauswaldt, Omar Jiménez Vázquez, Christian Utpatel, Stefan Niemann, Jan Rupp, Matthias Merker

**Affiliations:** 1 Molecular and Experimental Mycobacteriology, Research Center Borstel, Borstel, Germany; 2 German Center for Infection Research (DZIF), Partner Site Hamburg-Lübeck-Borstel-Riems, Hamburg, Germany; 3 Department of Infectious Diseases and Microbiology, University Hospital Schleswig-Holstein, Lübeck, Germany; 4 German Center for Infection Research (DZIF), TTU HAARBI, Lübeck, Germany; 5 Evolution of the Resistome, Research Center Borstel, Borstel, Germany

**Correction to: Genome Medicine 14, 13 (2022)**

**https://doi.org/10.1186/s13073-022-01017-x**

Following publication of the original article [1], the authors identified an error in Figs. 2 and 3. The correct figures are given below.

**Fig. 2:**
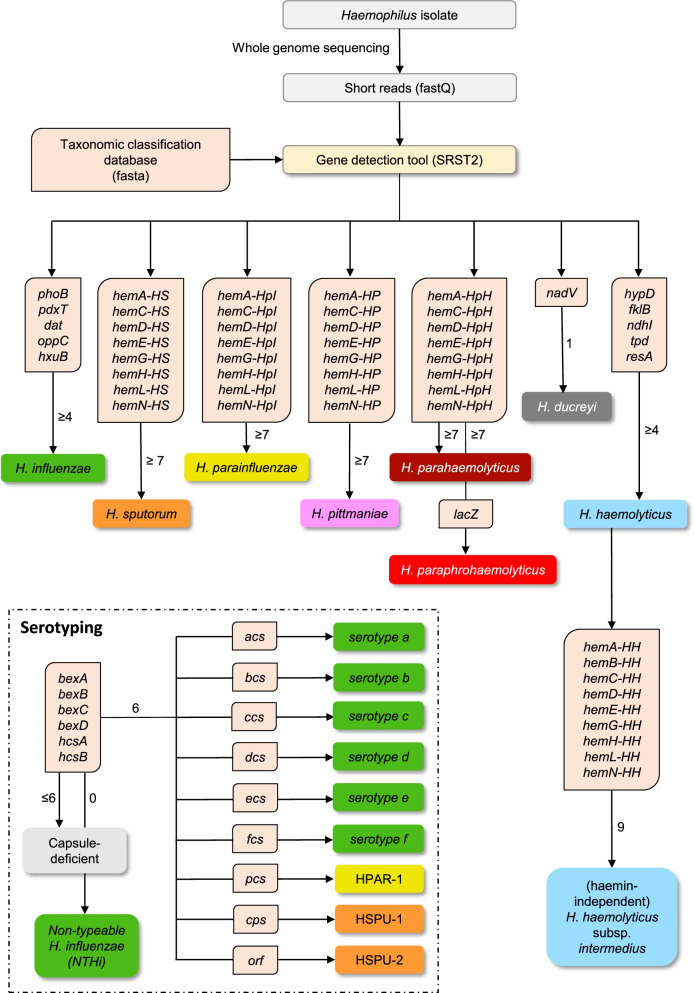
Decision algorithm to classify human-related strains of *Haemophilus* spp. based on whole genome sequencing data. The number next to the arrow specifies the minimum number of marker genes that needs to be detected before a (sub) species tag is attributed to the strain

**Fig. 3:**
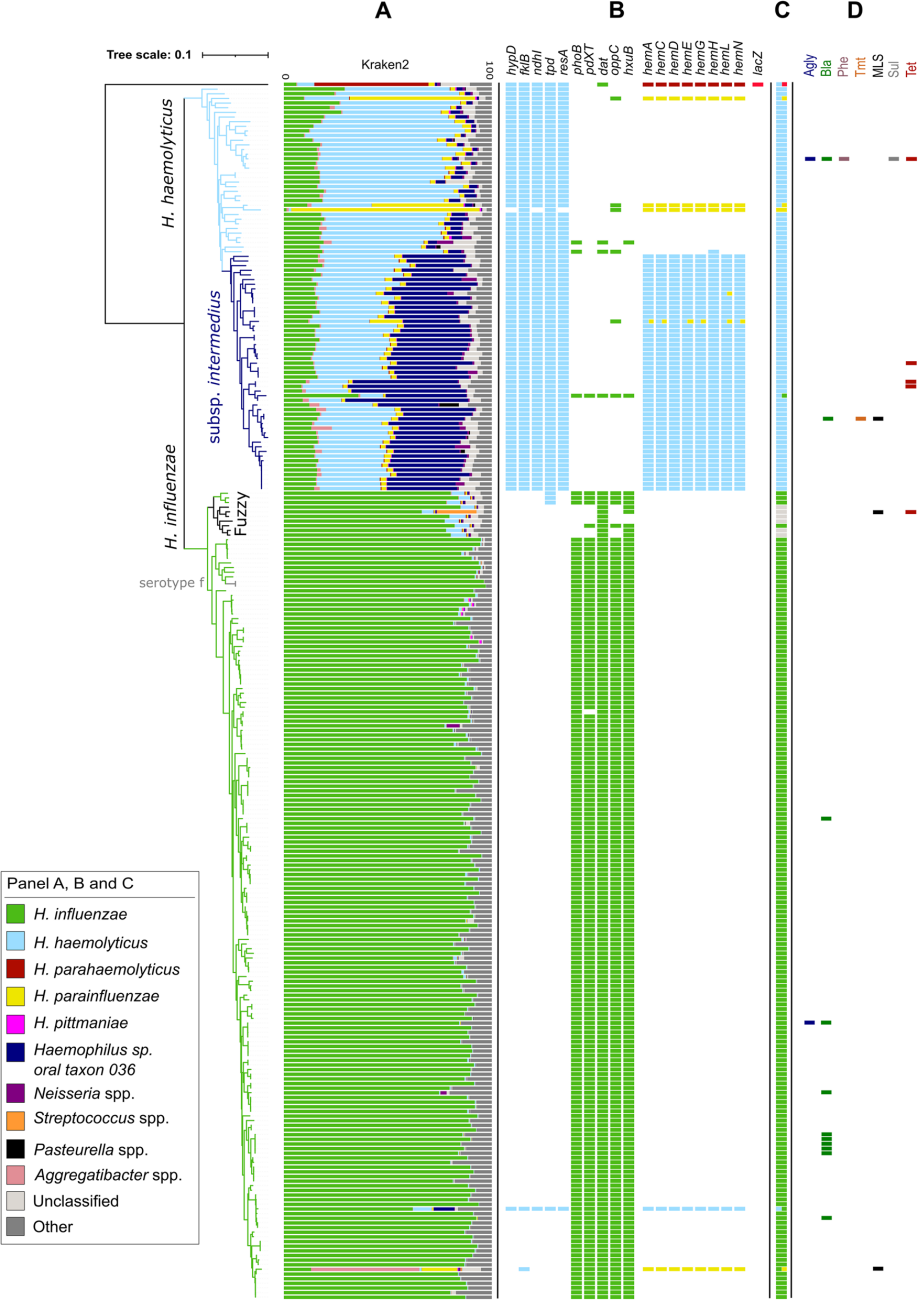
Phylogeny of 262 clinical *Haemophilus* spp. isolates from a German cohort. The phylogenetic tree is based on the alignment of 104 core genes (present in at least 90% of the strains). **A** Kraken2 read classification output. The length of a bar is proportional to the percentage of reads that are assigned to the respective taxon (as indicated by the color). One *H. influenzae* culture (located in the phylogenetic tree in the “fuzzy” clade) was likely contaminated with a *Streptococcus* sp. strain (19% of the reads assigned to this species) and another one with an *Aggregatibacter* sp. strain (52% reads assigned to this species). **B** Presence/absence of marker genes included in our new taxonomic classification database. **C** Final classification output of the decision algorithm. Mixed colors represent the presence of multiple full marker patterns, indicating multiple distinct *Haemophilus* species. **D** Presence/absence of antibiotic resistance genes included in a public resistance database. Color codes correlate to the antibiotic class to which the gene confers resistance: aminoglycosides (Agly), β-lactam antibiotics (Bla), phenicols (Phe), trimethoprim (Tmt), macrolide-lincosamide-streptogramin (MLS), sulfonamides (Sul), and tetracyclines (Tet)

The original article [1] has been corrected.
