# Supplementary material for: Whole genome sequencing-based classification of human-related Haemophilus species and detection of antimicrobial resistance genes
Source: Genome Med. 2022 Feb 9;14:13. doi: 10.1186/s13073-022-01017-x (PMC8830169; doi:10.1186/s13073-022-01017-x)
Supplement: Supplementary file 1 — Additional file 1: Table S1. Summary of the number of short read datasets used for each species in the training set, evaluation set, and German cohort. [file 13073_2022_1017_MOESM1_ESM.docx]

**Table S1 Summary of the number of short read datasets used for each species in the training set, evaluation set, and German cohort.** Classification is based on our new classification tool. ^1^ Five samples reported as *H. influenzae* at SRA were reclassified by our algorithm as *H. haemolyticus* or *H. parainfluenzae*. ^2^ All samples (n=262) were initially classified as *H. influenzae* at time of diagnosis (between 2008-2013). All *H. quentini* and *H. aegyptius* strains were classified by our algorithm as *H. haemolyticus* and *H. influenzae* by our algorithm, respectively.

| **Species/set** | **Training** | **Evaluation^1^** | **German cohort^2^** |
| --- | --- | --- | --- |
| ***H. influenzae*** | 68 | 1055 | 166 |
| ***NTHi*** | 53 | 521 | 164 |
| ***Serotypeable*** | 15 | 521 | 2 |
| ***Capsule-deficient*** | 0 | 11 | 0 |
| ***Multiple serotypes*** | 0 | 2 | 0 |
| ***H. (influenzae) aegyptius*** | 5 | 0 | 0 |
| ***H. haemolyticus*** | 61 | 125 | 83 |
| ***subsp. intermedius*** | 13 | 3 | 38 |
| ***H. (haemolyticus) quentini*** | 3 | 0 | 0 |
| ***H. parainfluenzae*** | 41 | 144 | 1 |
| ***Serotypeable*** | 0 | 11 | 0 |
| ***Capsule-deficient*** | 0 | 1 | 0 |
| ***H. parahaemolyticus*** | 8 | 0 | 0 |
| ***H. paraphrohaemolyticus*** | 4 | 2 | 0 |
| ***H. ducreyi*** | 17 | 3 | 0 |
| ***H. sputorum*** | 5 | 0 | 0 |
| ***Serotypeable*** | 5 | 0 | 0 |
| ***H. pittmaniae*** | 3 | 0 | 0 |
| ***Mix*** | 0 | 0 | 6 |
| ***Unknown*** | 0 | 0 | 6 |
| ***Total*** | 215 | 1329 | 262 |
