# Supplementary material for: Whole genome sequencing-based classification of human-related Haemophilus species and detection of antimicrobial resistance genes
Source: Genome Med. 2022 Feb 9;14:13. doi: 10.1186/s13073-022-01017-x (PMC8830169; doi:10.1186/s13073-022-01017-x)
Supplement: Supplementary file 6 — Additional file 6: Figure S1. Genomic arrangement of selected marker genes. [file 13073_2022_1017_MOESM6_ESM.docx]

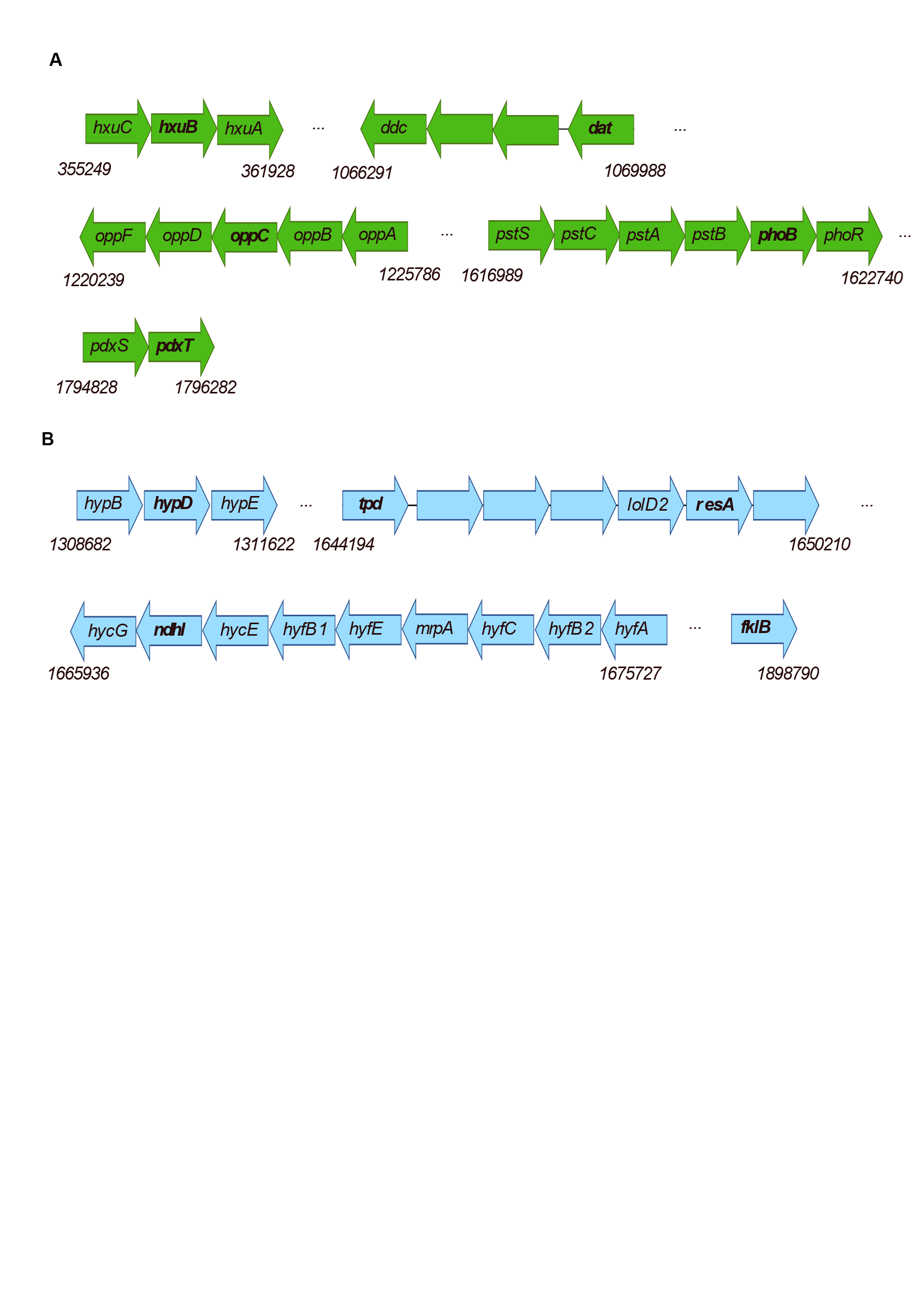


**Fig. S1: Genomic arrangement of selected marker genes.** A. *H. influenzae* marker genes with nucleotide numbering based on assembly NC_007146.2 (Strain 86-028NP). B. *H. haemolyticus* marker genes with nucleotide numbering based on assembly NZ_LS483458 (strain NCTC10839). The genes selected for the classification database are highlighted in bold. Other marker genes that were identified in the panGWAS analysis and were in close proximity to the selected genes are also visualized. Large distances between gene clusters are displayed by three dots. The length of an arrow is not proportional to the gene length.
