# Supplementary material for: Whole genome sequencing-based classification of human-related Haemophilus species and detection of antimicrobial resistance genes
Source: Genome Med. 2022 Feb 9;14:13. doi: 10.1186/s13073-022-01017-x (PMC8830169; doi:10.1186/s13073-022-01017-x)
Supplement: Supplementary file 7 — Additional file 7: Figure S2. Phylogenetic tree comprising training set strains and misclassified strains from SRA. [file 13073_2022_1017_MOESM7_ESM.docx]

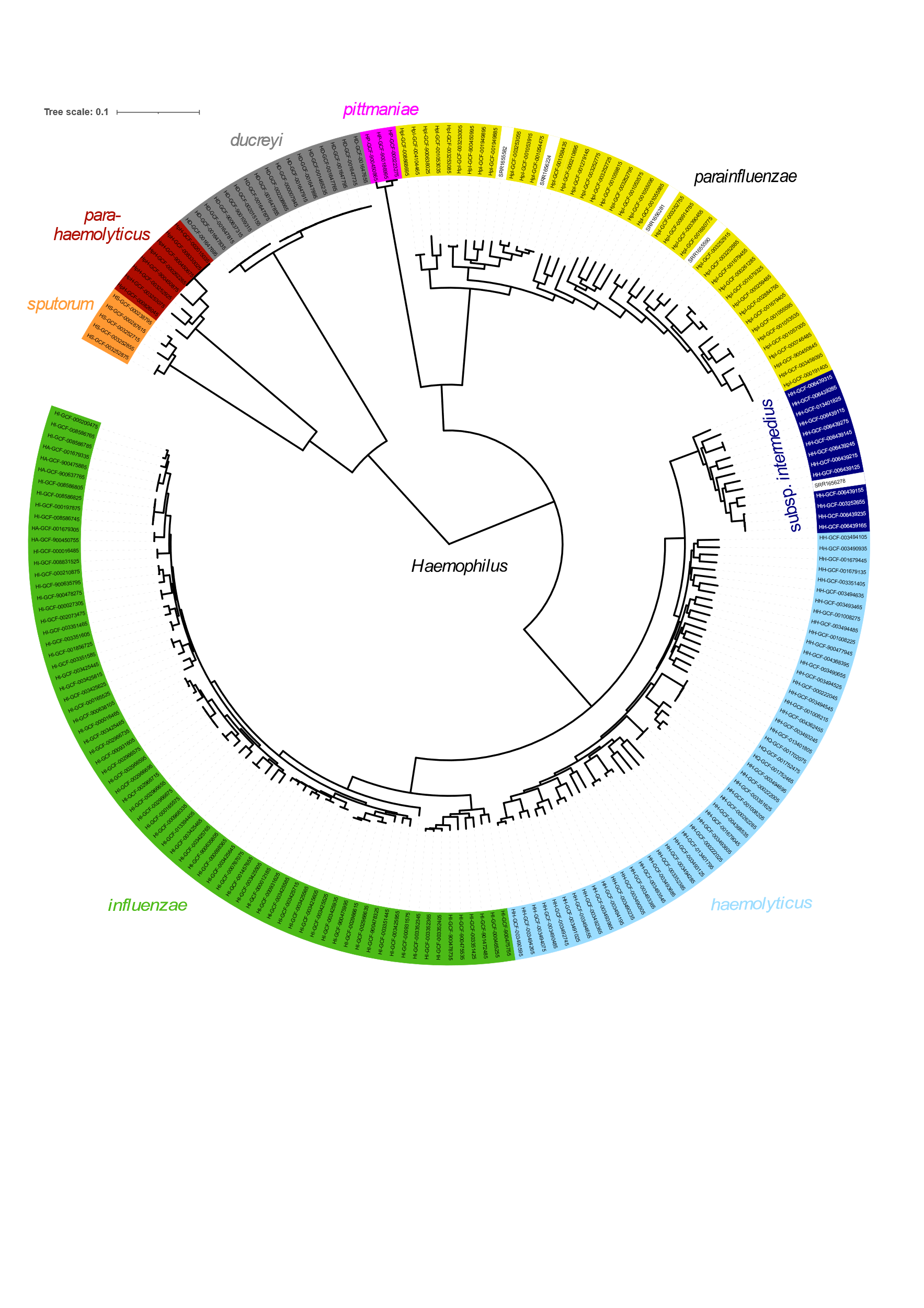


**Fig. S2: Phylogenetic tree comprising training set strains and misclassified strains from SRA.** The tree is based on the alignment of 935 core genes (present in at least 90% of the isolates) inferred from 211 whole genome sequencing (WGS) datasets of human related *Haemophilus* species downloaded from RefSeq (label colored according to species) and 5 WGS datasets downloaded from SRA (label not colored) which were annotated as *H. influenzae* and reclassified by our classification algorithm as *H. parainfluenzae* (n=4) or *H. haemolyticus* subsp. *intermedius* (n=1). The core genome was calculated with Roary (blastP 60% and splitting of paralogues parameter was turned off).
